# Notice to the Public

**Published:** 1870-04

**Authors:** 


### Notice to the Public.

We are happy to announce to the citizens of Buffalo that a most unblushing and ignorant quack, who last season fleeced them of their money and then obtained certificates of having cured them of diseases they never had, taking his pay in advance and at fabulous prices, has again returned. He may be consulted as formerly at all hours, and cures all diseases regarded incurable by regular physicians, gives no medicine, but by miraculous inherent curative qualities is enabled to relieve by a few sittings the most obstinate cases. A few respectable and well known families, who have nothing at all the matter, will be treated gratuitously for the consideration of a "*testimonial.*" Irregular practitioners having obstinate cases, are invited to hold consultations with him; to a few such the great secret of his cure will be explained. The daily press are also notified that should his stay be prolonged and prove usually remunerative, editorial (?) puffs will be paid for in cash. Copy furnished by the Doctor's own pen.

